# Dissecting the Sublexical Route for Reading: Frontal and Parietal Networks Support Learned Orthography-to-Phonology Mappings

**DOI:** 10.1162/NOL.a.216

**Published:** 2026-01-28

**Authors:** Sara M. Dyslin, Andrew T. DeMarco, Ryan Staples, J. Vivian Dickens, Sarah F. Snider, Rhonda Friedman, Peter E. Turkeltaub

**Affiliations:** Georgetown University Medical Center, Washington, DC, USA; Department of Rehabilitation Medicine, Georgetown University Medical Center, Washington, DC, USA; Department of Neurology, Georgetown University Medical Center, Washington, DC, USA; MedStar National Rehabilitation Hospital, Washington, DC, USA

**Keywords:** alexia, aphasia, dyslexia, lesion-symptom mapping, phonology, reading

## Abstract

Oral reading relies on lexical and sublexical processes with distinct neural mechanisms. Damage within the sublexical system causes phonological alexia, a blanket diagnosis describing acquired deficits in reading unfamiliar words. Improving the precision of alexia diagnosis requires understanding the neurocognitive basis of specific reading subprocesses. This study investigated the neural correlates of sublexical reading in 64 adults with chronic left-hemisphere stroke (LHS), focusing on lesions that impair the use of learned orthography-to-phonology (OP) mappings to read new words. Participants read aloud real words and three types of pseudowords varying in the number of plausible OP mappings at the level of the orthographic body: zero mappings (0M), one mapping (1M), and multiple mappings (MM). LHS participants exhibited phonological reading deficits with an exaggerated lexicality effect compared to 71 neurotypical controls. Across both groups, pseudowords with learned OP mappings were read more accurately than those without. Voxelwise and connectome-based lesion-symptom mapping revealed that relative lexical reading deficits were associated with lateral temporal lesions, while sublexical reading deficits were associated with lesions or disconnections of the left inferior frontal (IFG), supramarginal, and pre/postcentral gyri. Applying learned OP mappings relied on anterior IFG and frontoparietal connections, while resolving multiple plausible OP mappings relied on intraparietal connections. These results underscore the role of learned mutigraphemic OP mappings in sublexical reading, and demonstrate that disruptions of different sublexical reading subprocesses result in subtly different deficit patterns. Dissecting the neurocognitive basis of reading subprocesses may improve the precision of alexia diagnosis and point to new treatments.

## INTRODUCTION

Reading is an acquired skill that is fundamental for engagement in contemporary society. Among individuals who experience language impairments following left-hemisphere stroke (LHS), approximately two-thirds report specific challenges with reading, known as alexia, which significantly diminish independence and quality of life (Brookshire et al., 2014; Purdy et al., 2019). To better understand alexia and contribute to improved diagnosis and treatment planning, it is essential to understand both the cognitive and neural bases of reading.

Reading hinges on the dynamic interplay among three key types of knowledge: orthography (i.e., spelling), phonology (i.e., sound), and semantics (i.e., meaning) of written words. Central to all leading cognitive models of reading is the recognition of at least two pathways facilitating successful oral reading (Coltheart, 2006; Coltheart et al., 2001; Perry et al., 2007; Plaut et al., 1996). The lexical pathway supports the accurate pronunciation of known words, relying on whole-word mappings of orthography to phonology, potentially influenced by associated semantic content. Conversely, the sublexical pathway provides a direct link from orthography to phonology, aiding the decoding of letter combinations to their corresponding sounds. This pathway facilitates the pronunciation of novel words and pseudowords which lack stored lexical and semantic content. Compelling evidence for the distinction between these pathways emerges from alexia research, where dissociable syndromic patterns of alexia correspond to the lexical and sublexical routes. For example, phonological alexia is defined by a selective impairment of pseudoword reading relative to word reading that has been interpreted as an impairment to the sublexical pathway in the framework of reading models (Beauvois & Dérouesné, 1979).

Prior research into the mechanisms underlying sublexical reading has proposed that readers can either determine the pronunciation of novel words through orthography-to-phonology (OP) decoding or by using analogies to known words, which may involve indirect activation of lexical-semantic knowledge (Andrews & Scarratt, 1998; Patterson & Lambon Ralph, 1999). Given English’s orthographic inconsistencies, the letter groups that correspond to the orthographic body or syllable rime (vowel nucleus plus an optional consonant cluster in the coda) are especially utilized in spelling-to-sound translation (Bowey, 1990; Treiman et al., 1995). Careful orthotactic manipulations at the level of these subword OP mappings, specifically of the orthographic body, can therefore be useful to isolate mechanisms involved during different types of sublexical reading. For example, the orthographic body of pseudowords can be regular with only one plausible OP mapping (1M pseudowords; e.g., “bink” pronounced like “wink”), irregular with multiple plausible OP mappings (MM pseudowords; e.g., “tave,” which can be pronounced like “have” or “gave”), or unique with no analogous orthographic bodies in English (0M pseudowords; e.g., “dofe”; Andrews & Scarratt, 1998; Taft, 1982). It remains unclear to what extent these mappings at the level of the orthographic body (hereafter referred to as learned OP mappings) facilitate oral reading of novel words, and whether different types of OP mappings rely on distinct cognitive and neural substrates. Based on this framework, reading of 1M and MM pseudowords should rely on learned mappings at the level of the orthographic body, followed by assembly with the orthographic onset into a plausible pronunciation of the pseudoword. In contrast, 0M pseudowords without orthographic body neighbors should rely only on learned mappings of grapheme sequences at a smaller grain size, followed by assembly of those smaller units. Research on oral pseudoword reading has also suggested a consistency effect in which MM pseudowords may elicit slower reading speeds compared to 1M pseudowords (Glushko, 1979; Taraban & McClelland, 1987), potentially due to the supplemental involvement of semantic and lexical processes or additional cognitive demand required to disambiguate between multiple plausible pronunciations. This may reflect the influence of frequency-weighted probabilistic production mechanisms within the sublexical route (Zevin & Seidenberg, 2006), underscoring the need for further research to clarify these processes and their neural underpinnings.

Understanding the neural substrates that support these cognitive processes is crucial for advancing our knowledge of reading mechanisms. Functional neuroimaging studies, primarily in neurotypical adults, have delineated the general neural organization of the reading network, which includes a dorsal, phonological pathway facilitating assembly and articulation during reading, and a ventral, lexical-semantic pathway enabling access to lexical knowledge, including word meanings (Coltheart et al., 2001; Dehaene, 2009; Taylor et al., 2013; Turkeltaub et al., 2002). These pathways generally correspond to the sublexical and lexical pathways described in cognitive models of reading (Taylor et al., 2013). Lesion studies following LHS have further corroborated this distinction, highlighting correlations between lesion locations and deficits within each pathway (Coltheart et al., 2001; Dickens et al., 2019; Taylor et al., 2013). The dorsal sublexical pathway is typically isolated using a lexicality effect, comparing pseudoword reading to real word reading, either in terms of increased activity during functional magnetic resonance imaging (fMRI) studies or behavioral deficits in lesion studies. This contrast is effective in isolating sublexical processes, as it controls for speech production and subtracts out lexical-semantic processing, thus highlighting mechanisms unique to sublexical processing.

In comparison to real word reading, pseudoword reading elicits greater activation of several key brain regions, including the left posterior fusiform gyrus, left inferior parietal cortex, left inferior frontal gyrus (IFG), left precentral gyrus, and left insula (Binder et al., 2003; Carreiras et al., 2007; Dietz et al., 2005; Fiez et al., 1999; Graves et al., 2010; Hagoort et al., 1999; Mechelli et al., 2003, 2007; Taylor et al., 2013). Researchers frequently attribute OP translation to the inferior parietal cortex, as it consistently shows increased activity for pseudoword reading tasks, spelling and rhyming tasks, and training on novel symbol–phoneme associations (Baldo & Dronkers, 2006; Hashimoto & Sakai, 2004). The left IFG, insular cortex, and precentral gyrus have also been implicated in phonological processing above and beyond general executive demands that are not specific to reading (Binder et al., 2005; Mechelli et al., 2007; Poldrack et al., 1999). Specifically, the left IFG and insula have been proposed to correspond with related but different components of the cognitive models, namely, the phoneme system of the dual-route cascaded model, the phonological output buffer within connectionist dual process+ models, and the phoneme units within the triangle model (Coltheart et al., 2001; Perry et al., 2007; Taylor et al., 2013, respectively). A recent study of individuals with post-stroke alexia isolated brain regions corresponding to two different types of phonological impairment across several tasks, providing evidence for subspecialized regions within the sublexical route, with the inferior parietal cortex involved in sensorimotor translation and the ventral premotor cortex in motor phonology (Dickens et al., 2021). Thus, functional imaging and lesion studies have helped to delineate the types of phonological processing performed by different processing regions within the dorsal sublexical processing stream. Despite behavioral evidence that learned OP mappings at the level of the orthographic body are important for sublexical reading, the neural substrates underlying the use of these mappings for sublexical reading have not been examined.

In addition to identifying specific cortical regions involved in sublexical processing, diffusion MRI has enabled the study of the white matter pathways that support communication between these regions. Prior studies using diffusion tensor imaging with typical readers and individuals with developmental dyslexia have demonstrated that white matter pathways—including dorsal and ventral tracts such as the arcuate fasciculus, inferior longitudinal fasciculus, and uncinate fasciculus—are reliably associated with individual differences in reading skill and support both lexical and sublexical processing (Cummine et al., 2015; Ekstrand et al., 2020; Vandermosten et al., 2012). Structural connectome analysis uses tractography-based reconstructions of white matter connections to quantify the integrity of brain networks and has proven valuable in understanding how disconnections, in addition to focal lesions, contribute to language deficits after stroke (Dickens et al., 2021; McCall et al., 2022; Thiebaut de Schotten et al., 2020). Unlike functional connectome analyses that measure statistical dependencies in neural activity, structural connectome analysis characterizes the physical architecture of white matter pathways and can provide insights into how disruption of specific tracts impacts cognitive functions such as reading. This approach has been increasingly applied in post-stroke aphasia research and offers a complementary perspective to voxel-based lesion-symptom mapping by capturing the distributed nature of language processing and the role of network-level communication. Dickens et al. (2021) demonstrated that both voxel-based and disconnectome-based lesion-symptom mapping converged to implicate the left ventral precentral gyrus in motor-phonological impairments and reduced sublexical reading accuracy, and the left temporoparietal cortex in impaired sensory-motor integration and lower overall reading accuracy. However, the role of these pathways in applying learned OP mappings in reading has not yet been established.

In the current study, we aimed to bridge the gap in understanding the neural mechanisms of sublexical reading by (1) replicating and extending previous findings that lesions and disconnections in the dorsal phonological pathway result in sublexical reading deficits and (2) isolating the specific regions within the sublexical pathway that facilitate the application of learned OP mappings during pseudoword reading. To achieve this, LHS survivors and demographically matched control subjects completed oral reading tasks involving both real words and pseudowords manipulated according to the number of learned OP mappings at the level of the orthographic body. We compared behavioral performance across groups and stimulus types, and then used support vector regression voxel-based lesion-symptom mapping (SVR-VLSM) and connectome-based LSM (SVR-CLSM) analyses to identify lesion locations and disconnections associated with sublexical reading deficits. Specifically, we aimed to isolate lesions leading to deficits in the use of learned OP mappings during sublexical reading, selection among multiple OP mappings, and sublexical reading in the absence of learned OP mappings at the level of the orthographic body. Our findings provide critical evidence for distinct neural substrates underlying sublexical and lexical reading, and that learned OP mappings facilitate sublexical reading via dissociable neural subprocessors within the sublexical pathway.

## MATERIALS AND METHODS

### Participants

Participants included 64 adults with a history of LHS and 71 neurotypical controls (see Table 1). Participants were native English speakers with no significant neurological or neuropsychiatric disorders other than stroke. Sixty-one LHS participants were in the chronic stage of stroke recovery (i.e., at least 6 months post-stroke), and three additional participants were included in the subacute stage (2.3, 3.9, and 4.0 months post-stroke). Control participants with no history of stroke were matched to the stroke group on age and education. All participants provided written informed consent, and the Georgetown University Institutional Review Board approved the study protocol. Participants were recruited as a part of a larger study on post-stroke language outcomes (Clinicaltrials.gov NCT04991519). LHS participants were excluded if they could not complete the oral reading tasks (at least 5/200 real words). Years of education were determined based on the highest degree attained in the United States (high school = 12, college = 16, master’s = 18, JD = 19, MD = 20, PhD = 21). Participants without a high school degree were assigned a value corresponding to the highest grade level completed. Overall aphasia severity was assessed with the Western Aphasia Battery Aphasia Quotient (WAB-AQ), a summary score ranging from 0 to 100, with lower scores indicating more severe language impairment. A score below 93.8 is typically used as the clinical cutoff for aphasia (Kertesz, 2006), although many people with mild aphasia score above this cutoff (Laks et al., 2025). According to WAB–Revised classifications, the breakdown of aphasia subtypes within our sample are as follows: 46 anomic, 8 conduction, 4 Broca’s, 4 transcortical motor, and 2 Wernicke’s.

**Table 1.** Participant characteristics

	Controls (*n* = 71)	LHS (*n* = 64)
Sex	35 F, 36 M	29 F, 35 M
Handedness	66 R, 5 L	57 R, 6 L, 1 A
Age (yr)	61.6 (11.5), range: 31–83	61.6 (11.6), range: 39–92
Race	24 African American, 47 Caucasian	24 African American, 42 Caucasian
Ethnicity	69 Non-Hispanic/Latino, 2 unknown	3 Hispanic/Latino, 61 Non-Hispanic/Latino
Education	17.2 (2.5), range: 12–21	16.5 (2.8), range: 9–21
Stroke chronicity (mo)	–	49.9 (55.1), range: 2.3–269.7
Mean lesion volume (cubic cm)	–	90.2 (78.2), range: 0.7–349.3
Western Aphasia Battery Aphasia Quotient	–	82.3 (15.8), range: 32.2–99.6

*Note*. Demographic information is listed for both groups as well as stroke and aphasia characteristics for left hemisphere stroke (LHS) participants. Handedness is self-reported as left (L), right (R), or ambidextrous (A). Parentheses indicate *SD*.

### Oral Reading Assessments

Participants read aloud a list of 200 real words and 60 pseudowords. Real word oral reading included 200 monosyllabic words that varied orthogonally in frequency, spelling-to-sound regularity, and imageability (Staples et al., 2025). Three types of pseudowords were equally presented (20 stimuli each), differentiated based on the number of OP body mappings that exist in English: zero mappings (0M), one mapping (1M), and multiple mappings (MM). 0M pseudowords (e.g., “dofe”) are pseudowords whose orthographic bodies do not exist within the English lexicon. 1M pseudowords (e.g., “bink”) have only one plausible pronunciation based on orthographic bodies in English (i.e., -ink as in “sink”). MM pseudowords (e.g., “chead”) involve at least two plausible pronunciations based on existing orthographic bodies in English (i.e., -ead pronounced either as in “bead” or “head”). Pseudoword stimuli were adapted and extended from Friedman et al. (1992; see Supplemental Table S2, available at https://doi.org/10.1162/NOL.a.216). Pseudowords were matched to words on body consistency, positional bigram frequency, and articulatory complexity (Stoel-Gammon, 2010). All stimuli were monosyllabic and 3–6 letters in length.

Participants were seated at a table in a quiet testing room with a 17″ Dell Inspiron touch-screen laptop in tent-mode. The tasks were administered in E-Prime 3.0. Real words were presented in blocks of 25 items with breaks between blocks. Pseudowords were presented in blocks of 20 items, with pseudoword types randomized within blocks. To avoid priming effects from real word stimuli, the pseudoword reading task was administered first, followed by the real word reading task later in the testing sessions. Both the order of task administration and the order of items within each task were held constant across participants. The tasks were self-paced, and each trial timed out if the participant did not advance to the next item after 10 s. The total time spent on each item was recorded as a proxy for reading duration, reflecting how long participants engaged with each oral reading item. Relative to standard reaction time measures, this metric captures a broader range of reading-related processes, including phonological retrieval, motor planning, and articulation. These components may be particularly relevant for distinctions within sublexical reading and the application of learned OP mappings. Responses were video recorded and scored for accuracy offline. Scoring was completed based on the first complete reading attempt containing both a consonant and a vowel, and any response that included a plausible pronunciation of the presented pseudoword or real word was scored as correct. Responses to pseudowords were coded as incorrect if they contained spelling-to-sound mappings that do not occur in American English.

### Behavioral Analyses

Two logistic mixed effect models were estimated to determine the effects of group (LHS vs. controls), lexicality, and pseudoword type on reading accuracy. The first model assessed the main effects of group and lexicality on reading accuracy, as well as their interaction (group by lexicality), while including fixed effects of age and education level and random effects of subject and item. The second model assessed the main effects of group and pseudoword type on pseudoword reading accuracy, as well as their interaction (group by pseudoword type), while including fixed effects of age and education level and random effects of subject and item. Pseudoword type was dummy coded within the model with 0M pseudowords serving as the reference category.

Four additional mixed effects models were estimated to determine the effects of lexicality and pseudoword type on reading accuracy within each group with the same fixed effects of age and education level and random effects of subject and item. All models were fitted using the glmer function in R Statistical Software with maximum likelihood estimation (Version 4.1.2; R Core Team, 2021). Significance levels were determined at *p* < 0.05. Results of the logistic mixed effects model are reported as odds ratios and associated 95% confidence intervals, with statistical significance determined through the Wald *Z*-statistic and its associated *p* value.

Two additional linear mixed effects models were estimated within the control group to examine the effects of lexicality and pseudoword type on reading duration, operationalized as the total time spent on each item. An inverse measure of this duration (1/time on item) was used as the dependent variable to normalize the skewed distribution. Both models included fixed effects of age and education level, and random intercepts for subject and item. The first model examined the effect of lexicality (real words vs. pseudowords), and the second model examined differences in reading duration among the three pseudoword types, with 0M pseudowords serving as the reference category. Models were fitted using the lmer function in R (Version 4.1.2; R Core Team, 2021) with restricted maximum likelihood estimation. Significance was determined using Satterthwaite’s method for degrees of freedom (via the lmerTest package), with a threshold of *p* < 0.05.

### Neuroimaging

#### Structural imaging acquisition

All brain images were acquired on Georgetown University’s 3T Siemens Prisma scanner. Scans of each participant included a T1-weighted magnetization prepared rapid gradient echo (MPRAGE), a fluid-attenuated inversion recovery (FLAIR) sequence, and diffusion imaging sequence. High resolution T1-weighted images were acquired for all participants with the following parameters: 176 sagittal slices; slice thickness = 1 mm, 1 mm^3^ voxels; field of view (FOV) = 256; matrix = 256 × 256; flip angle = 9°; generalized autocalibrating partial parallel acquisition = 2; repetition time (TR) = 1,900 ms; echo time (TE) = 2.98 ms; scan time: ∼5 min. The FLAIR sequence aided in manual lesion tracing and was acquired with the following parameters: 192 sagittal slices, slice thickness = 1 mm; 1 mm^3^ voxels; flip angle = 120°; FOV = 256 mm, matrix = 256 × 256; TR = 5,000 ms; TE = 386 ms; inversion time (TI) = 1,800 ms; scan time: ∼6 min. Multishell high angular diffusion imaging scans were acquired to quantify white matter connections with the following parameters: MS-HARDI; 74 axial slices; slice thickness = 2 mm; diffusion-weighted gradients; 81 directions at *b* = 3,000, 40 at *b* = 1,200, 7 at *b* = 0, 70 slices; 2 mm cubic voxels; flip angle = 90°; phase encoding direction = anterior to posterior; partial Fourier = 6/8; FOV = 232 mm; matrix = 116 × 116; TR = 5.0 s; TE = 0.082 s; readout time = 0.061 s; slice acceleration = 1; scan time: ∼10 min. Six reverse phase-encoded *b* = 0 images were acquired for susceptibility field estimation (scan time: ∼1 min).

#### Lesion tracing and normalization

Stroke lesions were manually segmented on each participant’s MPRAGE and FLAIR images using ITK-SNAP software and approved by P.E.T. MPRAGEs and lesion masks were warped to the Clinical Toolbox Older Adult Template using Advanced Normalization Tools (ANTs; https://stnava.github.io/ANTs/) through a custom standard pipeline described elsewhere (Avants et al., 2011; Martin et al., 2025).

#### Structural connectome construction

Structural connectomes were derived from MS-HARDI scans which were preprocessed in MRtrix 3.0 (Tournier et al., 2019) according to methods described in Dickens et al. (2021). This pipeline included the standard stepwise application of Gaussian noise removal, Gibbs ringing artifact removal, correction of distortions induced by motion, eddy currents, and magnetic susceptibility, and inhomogeneity distortion correction. Fiber orientation distributions (FODs) were estimated using multishell, multi-tissue constrained spherical deconvolution with group-average response functions derived from a healthy control and stroke cohort (Dickens et al., 2021).

Structural connectivity was quantified through 15 million streamlines generated by probabilistic anatomically constrained tractography (Smith et al., 2012) on the white matter fiber orientation distributions in native space (algorithm = iFOD2, step size = 1 mm, min/max length = 10/300, maximum angle = 45, backtracking allowed, dynamic seeding, streamlines cropped at gray matter–white matter interface). Edges of the structural connectome were generated by assigning streamlines to parcels of the Lausanne atlas at scale 125 (https://github.com/mattcieslak/easy_lausanne; Daducci et al., 2012), yielding a 2D structural connectivity weighted adjacency matrix for each participant. Each edge in the connectome is proportional to the cross-sectional area of white matter connecting the two parcels at the intersecting row–column. Unlike fiber density or composite weighting approaches, using cross-sectional area allows for more robust detection of complete disconnections while reducing sensitivity to individual differences in connection strength.

We then create a disconnection matrix for each stroke survivor by labeling each connection between brain parcels (i.e., an edge) in the stroke survivors’ connectomes as lesioned if it was absent in a stroke survivor’s connectome but present in 100% of control subjects’ connectomes. This procedure yielded a binary disconnection matrix for each stroke survivor in which disconnected edges have a value of 1 and intact connections have a value of 0, analogous to a binary voxelwise lesion mask. This binary framework, based on tract absence rather than graded integrity, enhances interpretability in lesion-symptom mapping by anchoring network disruption to the presence or absence of structural connections.

#### Lesion overlap maps

**Figure 1.**
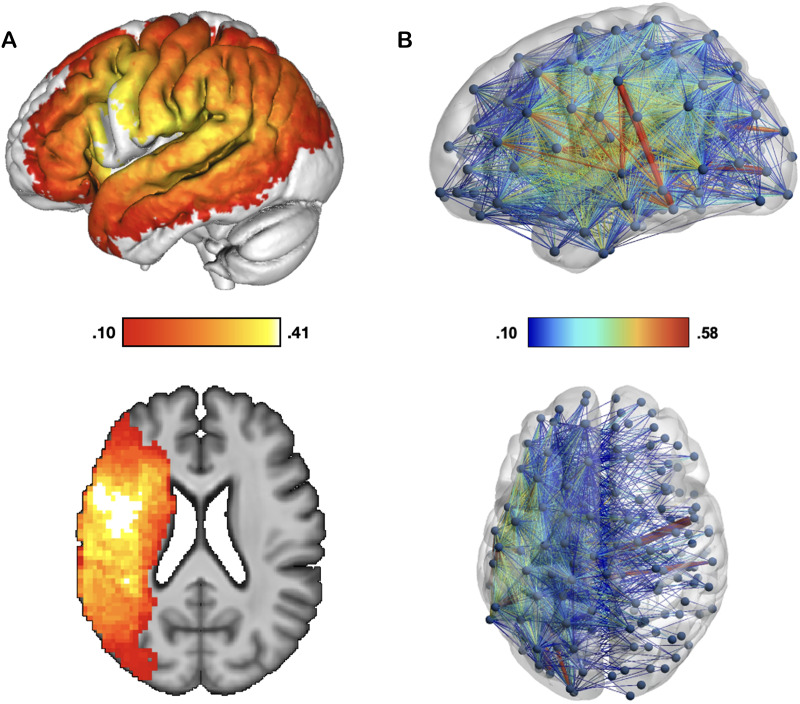
Voxel and connectome-based lesion overlaps. Lesion overlap maps are shown for the 64 participants with a history of left-hemisphere stroke: (A) voxelwise level; (B) connectome level. Color scales indicate the proportion of participants with lesions to those voxels and disconnections.

The overlap of the patients’ lesions reveals widespread damage predominantly in the perisylvian area, characteristic of strokes of the left middle cerebral artery (Figure 1A). The disconnectome overlap map shows a significant loss of interhemispheric connections and connections within the left hemisphere as expected (Figure 1B).

### Lesion-Symptom Mapping Analyses

To identify the brain regions subserving reading and sublexical processes facilitating the application of learned OP mappings, we conducted SVR-LSM at the voxelwise and connectome levels (Zhang et al., 2014). The SVR-VLSM analyses were conducted using a MATLAB toolbox developed by our group (DeMarco & Turkeltaub, 2018). Analyses were limited to voxels lesioned in at least 10% of participants to ensure sufficient statistical power and lesion coverage variance according to commonly adopted standards in the literature (Kimberg et al., 2007). Covariates of lesion volume, age, and education were regressed out of both the lesion and behavioral data prior to modeling.

Our SVR-CLSM approach is an extension of SVR-VLSM which quantifies lesion through anatomical disconnection and thus identifies parcel-based neuroanatomical networks, as opposed to voxel-based clusters. The SVR-CLSM analyses complement the SVR-VLSM analyses by identifying the necessary contributions of both lesioned and spared, but disconnected, brain regions to reading. Lesion volume, age, and education were regressed out of both the connectome edge values and behavioral data. Only edges at which greater than 10% of stroke participants had lesions (i.e., 0 edges after binarization) were included in the analysis. Connections not present in 100% of control subjects were excluded from analyses in order to reduce Type I error. Statistical significance of the SVR-CLSM beta-maps was determined via a permutation-based familywise error rate correction (FWER; 10,000 permutations, FWER *p* < 0.05, one-tailed [negative]; Kimberg et al., 2007) and was evaluated at both the edge-level (disconnection between pairs of brain parcels) and parcel-level (disconnection of a single brain parcel, including all anatomical endpoints).

We conducted five pairs of voxel-based and connectome-based SVR-LSM analyses to isolate lesion locations and disconnections associated with specific reading and sublexical processing deficits. First, we identified brain regions that are important for sublexical reading with SVR-LSM of accuracy on all pseudowords controlling for accuracy on all real words. This first contrast identifies lesion locations associated with an exaggerated lexicality effect (consistent with diagnoses of phonological alexia). Next, SVR-LSM was conducted for the reverse contrast, accuracy on all real words controlling for pseudowords, to identify lesions associated with the reduction of a lexicality effect.

Our next three critical analyses elaborated on sublexical processes. The first sublexical contrast aimed to isolate neural correlates important for the application of learned OP mapping during sublexical reading, by identifying lesion locations and disconnections associated with reading accuracy on pseudowords with existing OP mappings (MM and 1M) while covarying for two variables: accuracy on pseudowords with zero mappings (0M) and accuracy on real words. The second sublexical contrast aimed to isolate neural correlates underlying the sublexical processes important for accuracy on pseudowords with multiple plausible OP mappings (MM) covarying for 1M pseudowords and accuracy on real words. The final sublexical contrast aimed to isolate neural correlates important for sublexical processes given unfamiliar orthographic bodies and therefore absent of relevant learned OP mappings, specifically accuracy on 0M pseudowords controlling for accuracy on MM and 1M pseudowords and accuracy on real words.

## RESULTS

### Behavioral Results

**Figure 2.**
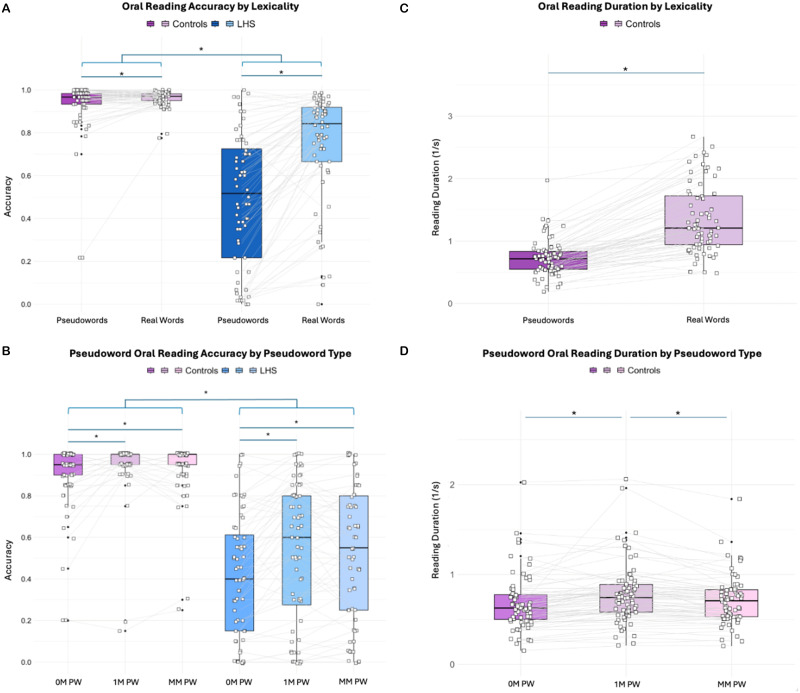
Oral reading accuracy by lexicality and pseudoword type. (A) The average accuracies across trials for real word oral reading and pseudoword oral reading (collapsed across pseudoword types) are shown for control participants (left) and participants with LHS (right). (B) The average accuracies on responses across trials for each pseudoword type within the oral pseudoword reading task are shown for control participants (left) and LHS participants (right). (C) The average reading durations across trials for real word oral reading and pseudoword oral reading are shown for control participants. The *y*-axis represents an inverse reading duration metric (1/time on item(s)); thus, higher values indicate faster overall responses. (D) The average reading durations across trials for each pseudoword type within the oral pseudoword reading task for control participants. White squares denote individual participants, and gray lines connect each individual’s average performance across the two tasks. Solid lines above the data distributions indicate significant main effects of group and lexicality (A), and pseudoword type (B). Bracketed lines indicate significant interactions between group and lexicality (A) or group and pseudoword type (B). See Table 2 for descriptive statistics. See Tables 3–6 for results of the mixed effect models predicting accuracy. See Table 7 for the results of mixed effects models predicting reading duration. LHS = left-hemisphere stroke, PW = pseudoword, 0M = zero mappings, 1M = one mapping, MM = multiple mappings.

LHS participants showed greater variability in performance compared to control participants, as expected (Figure 2 and Table 2). The first logistic mixed effect model showed an expected main effect of group (*Z* = −8.91, *p* < 0.001, OR = 0.070, 95% CI = [0.04, 0.13]) such that control participants read more accurately overall compared to LHS participants (Figure 2A and Table 3). There was also a main effect of lexicality (*Z* = −5.65, *p* < 0.001, OR = 0.337, 95% CI = [0.23, 0.49]) indicating that accuracy across groups was significantly higher for words compared to pseudowords. A significant interaction of group and lexicality was observed (*Z* = −8.79, *p* < 0.001, OR = 0.398, 95% [CI = 0.32, 0.49]), demonstrating an exaggerated lexicality effect, that is, phonological reading deficits, in LHS participants relative to controls.

**Table 2.** Descriptive statistics for oral reading accuracy in controls and LHS participants and oral reading duration (s) in controls

	Real words (*n* = 200)		All pseudowords (*n* = 60)		0M pseudowords (*n* = 20)		1M pseudowords (*n* = 20)		MM pseudowords (*n* = 20)	
	Controls	LHS	Controls	LHS	Controls	LHS	Controls	LHS	Controls	LHS
Accuracy										
Missing	3	1	1	0	1	0	1	0	1	0
Mean	94.3%	75.0%	93.0%	48.9%	89.7%	41.2%	94.3%	52.9%	93.2%	52.7%
*SD*	12%	24%	15%	31%	16%	30%	15%	34%	14%	33%
Min.	77.5%	0.09%	22%	0%	20%	0%	15%	0%	25%	0%
Max.	100%	98.5%	100%	100%	100%	100%	100%	100%	100%	100%
Duration (s)										
Missing	0		0		0		0		0	
Mean	0.98		1.75		1.88		1.62		1.73	
*SD*	0.60		1.05		1.17		0.99		0.97	
Min.	0.31		0.31		0.31		0.40		0.33	
Max.	9.09		9.85		9.85		9.06		9.21	

*Note*. LHS = left-hemisphere stroke.

**Table 3.** Results of a model examining lexicality and history of stroke to accuracy reading aloud

	*SD*	Odds ratios	95% CI	*p*	*p* < 0.05
Predictors					
Education	–	1.082	[0.97, 1.21]	0.159	
Age	–	1.015	[0.99, 1.04]	0.255	
Group	–	0.070	[0.04, 0.13]	<0.001	*
Lexicality	–	0.337	[0.23, 0.49]	<0.001	*
Group * Lexicality	–	0.398	[0.32, 0.49]	<0.001	*
Random effects					
Random intercept: Item	1.30	–			
Random intercept: Subject	2.76	–			
N_subject_	135	–			
N_item_	260	–			

*Note*. Formula: Accuracy ∼ 1 + Education by degree + Age + Group * Lexicality + (1|Subject) + (1|Item).

If learned OP mappings facilitate sublexical reading, then both groups should show increased accuracy for pseudowords with learned mappings (1M and MM pseudowords) relative to those without any orthographic body neighbors in English (0M pseudowords). Consistent with this hypothesis, a logistic mixed effect model revealed a significant main effect of pseudoword type on pseudoword reading accuracy, such that across groups 1M and MM pseudowords were read with increased accuracy relative to 0M pseudowords (Figure 2B; *Z* = 3.63, *p* < 0.001, OR = 2.58, 95% CI = [1.55, 4.29]; *Z* = 2.54, *p* = 0.011, OR = 1.90, 95% CI = [1.16, 3.12], respectively). Accuracy did not significantly differ between 1M and MM pseudowords. This model also showed a significant main effect of group such that LHS was related to reduced pseudoword reading accuracy (*Z* = −10.48, *p* < 0.001, OR = 0.337, 95% CI = [0.23, 0.49]). There were no significant interactions of group by pseudoword type. Full model estimates are shown in Table 4.

**Table 4.** Results of a model relating pseudoword type and history of stroke to accuracy reading aloud

	*SD*	Odds ratios	95% CI	*p*	*p* < 0.05
Predictors					
Education	–	1.12	[0.98, 1.28]	0.094	
Age	–	1.00	[0.97, 1.03]	0.784	
Group	–	0.021	[0.01, 0.04]	<0.001	*
1M vs. 0M pseudowords	–	2.58	[1.55, 4.29]	<0.001	*
MM vs. 0M pseudowords	–	1.90	[1.16, 3.12]	<0.001	*
Group * 1M vs. 0M pseudowords	–	0.85	[0.57, 1.27]	0.422	
Group * MM vs. 0M pseudowords	–	1.13	[0.77, 1.66]	0.541	
Random effects					
Random intercept: Item	0.36	–			
Random intercept: Subject	3.64	–			
N_subject_	133	–			
N_item_	60	–			

*Note*. Formula: Accuracy ∼ 1 + Education by degree + Age + Group * Pseudoword type + (1|Subject) + (1|Item).

Additional within-group mixed effect models were estimated to determine effects of lexicality and pseudoword type without group as a fixed effect (see Table 5 and Table 6). A main effect of lexicality was observed in both control and LHS participants such that real words were read with greater accuracy than pseudowords (controls: *Z* = −5.84, *p* < 0.001, OR = 0.165, 95% CI = [0.09, 0.30]; LHS participants: *Z* = −12.19, *p* < 0.001, OR = 0.143, 95% CI = [0.10, 0.20]). A main effect of pseudoword type was also observed in both control and LHS participants for both 1M and MM pseudowords relative to 0M pseudowords (see Table 6). As in the combined model, there were no significant differences in accuracy between 1M and MM pseudowords in either group. In both models, controls also revealed a significant main effect of education such that more prior education related to increased reading accuracy (see Table 5 and Table 6). The lexicality effect model in LHS participants revealed a significant main effect of age such that increased age related to higher reading accuracy (see Table 5 and Table 6).

**Table 5.** Results of two models relating lexicality to accuracy reading aloud within control participants and LHS participants

	*SD*	Odds ratios	95% CI	*p*	*p* < 0.05
Control participants					
Predictors					
Education	–	1.218	[1.08, 1.38]	0.002	*
Age	–	0.989	[0.96, 1.02]	0.384	
Lexicality	–	0.165	[0.09, 0.30]	<0.001	*
Random effects					
Random intercept: Item	3.25	–			
Random intercept: Subject	1.41	–			
N_subject_	70	–			
N_item_	260	–			
LHS participants					
Predictors					
Education	–	0.947	[0.79, 1.13]	0.548	
Age	–	1.048	[1.00, 1.09]	0.029	*
Lexicality	–	0.143	[0.10, 0.20]	<0.001	*
Random effects					
Random intercept: Item	1.04	–			
Random intercept: Subject	3.73	–			
N_subject_	65	–			
N_item_	260	–			

*Note*. Formula: Accuracy ∼ 1 + Education by degree + Age + Lexicality + (1|Subject) + (1|Item).

**Table 6.** Results of two models relating pseudoword type to accuracy reading aloud within control participants and LHS participants

	*SD*	Odds ratios	95% CI	*p*	*p* < 0.05
Control participants					
Predictors					
Education	–	1.320	[1.14, 1.53]	<0.001	*
Age	–	0.982	[0.98, 1.01]	0.265	
1M vs. 0M pseudowords	–	2.651	[1.54, 4.57]	<0.001	*
MM vs. 0M pseudowords	–	1.958	[1.15, 3.33]	<0.001	*
Random effects					
Random intercept: Item	0.41	–			
Random intercept: Subject	1.74	–			
N_subject_	69	–			
N_item_	60	–			
LHS participants					
Predictors					
Education	–	0.945	[0.77, 1.16]	0.593	
Age	–	1.022	[0.97, 1.07]	0.378	
1M vs. 0M pseudowords	–	2.190	[1.45, 3.31]	<0.001	*
MM vs. 0M pseudowords	–	2.155	[1.43, 3.26]	<0.001	*
Random effects					
Random intercept: Item	0.34	–			
Random intercept: Subject	4.88	–			
N_subject_	64	–			
N_item_	60	–			

*Note*. Formula: Accuracy ∼ 1 + Education by degree + Age + Pseudoword type + (1|Subject) + (1|Item).

To evaluate oral reading speed, two final linear mixed effects models were estimated in control participants to examine the effects of lexicality and pseudoword type on an inverse measure of reading duration (Figure 2C–D and Table 7). In the first model, a main effect of lexicality was observed, with pseudowords read more slowly than real words (*β* = 0.625, 95% CI = [0.588, 0.661], *p* < 0.001). The second model evaluated reading durations for pseudowords as a function of learned OP mapping frequency. Reading durations differed significantly by pseudoword type, consistent with the hypothesis that learned mappings support sublexical decoding. Specifically, pseudowords with one learned OP mapping (1M) were read more quickly than those without any (0M; *β* = −0.103, 95% CI = [−0.144, −0.062], *p* < 0.001). One-mapping pseudowords were also read more quickly than those with multiple potential OP mappings (MM; *β* = −0.070, 95% CI = [−0.111, −0.029], *p* = 0.001), whose varied pronunciations may introduce an additional layer of ambiguity. A direct comparison between 0M and MM pseudowords, using 0M as the reference level, revealed no significant difference (*p* = 0.115), suggesting comparable reading durations between these types. No significant effects of age or education were observed in either model of reading duration.

**Table 7.** Results of two models relating lexicality and pseudoword type to oral reading duration within controls

	*SD*	Estimate (*β*)	95% CI	*p*	*p* < 0.05
Control participants					
Formula: Reading duration ∼ 1 + Education by degree + Age + Lexicality + (1|Subject) + (1|Item)					
Predictors					
Education	–	−0.029	[−0.074, 0.017]	0.216	
Age	–	−0.005	[−0.014, 0.005]	0.349	
Lexicality	–	0.625	[0.588, 0.661]	<0.001	*
Random effects					
Random intercept: Item	0.12	–			
Random intercept: Subject	0.47	–			
N_subject_	70	–			
N_item_	260	–			
Control participants					
Formula: Reading duration ∼ 1 + Education by degree + Age + Pseudoword type + (1|Subject) + (1|Item)					
Predictors					
Education	–	−0.002	[−0.030, 0.027]	0.908	
Age	–	∼0.000	[−0.006, 0.006]	0.963	
0M v.s 1M pseudowords	–	−0.103	[−0.144, −0.062]	<0.001	*
MM vs. 1M pseudowords	–	−0.070	[−0.111, −0.029]	0.001	*
MM vs. 0M pseudowords	–	0.033	[−0.008, 0.074]	0.115	
Random effects					
Random intercept: Item	0.06	–			
Random intercept: Subject	0.29	–			
N_subject_	69	–			
N_item_	60	–			

### Lesion-Symptom Mapping

#### Lexicality contrasts

**Figure 3.**
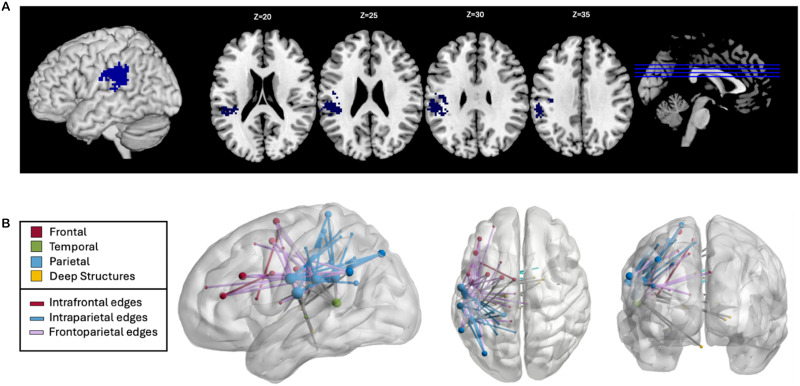
Lesion-symptom mapping for pseudowords controlling for real words. (A) Voxelwise lesion-symptom mapping results at *p* < 0.005 and clusterwise familywise error *p* < 0.05. (B) Connectome lesion-symptom mapping results show all significant edges color-coded by lobe, regardless of hemisphere. Edges that are not intrafrontal, intraparietal, or frontoparietal are shown in gray. Node size relates to the number of significant edges at that node.

**Figure 4.**
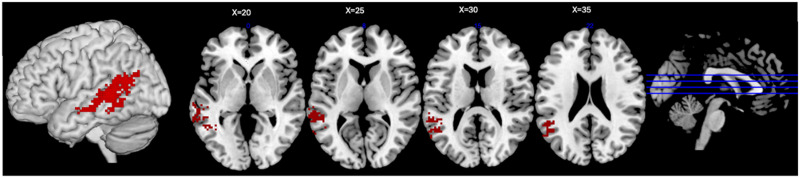
Lesion-symptom mapping for real words controlling for pseudowords. Voxelwise lesion-symptom mapping results at *p* < 0.005 and clusterwise familywise error *p* < 0.05.

Two SVR-LSM analyses were performed to investigate whether damage to specific brain regions predicted reduced oral reading accuracy of pseudowords and real words, each controlling for the other stimulus type. Lesions involving left supramarginal gyrus (SMG) related to reduced oral pseudoword reading accuracy (SVR-VLSM clusterwise *p* = 0.03; cluster size: 5.500 cm^3^; center of mass: −48.4, −30, 26; Figure 3A). Fifty-three disconnections similarly related to reduced oral pseudoword reading accuracy (SVR-CLSM edgewise FWER *p* < 0.05; see Figure 3B and Supplemental Table S1). Of these 53 disconnections, 17 connected left-hemisphere (LH) frontal and parietal regions, 17 were intraparietal connections, and 4 were interhemispheric connections. Several regions were particularly implicated by these findings, including the left postcentral gyrus, the left SMG, and the left middle frontal gyrus (MFG; involved in 31, 22, and 11 disconnections, respectively). The left precentral gyrus, left inferior parietal lobule, and left superior temporal sulcus were also each involved in six disconnections. Lesions to the middle temporal gyrus (MTG), superior temporal sulcus, and surrounding cortex and white matter resulted in the relative reduction of oral real word reading accuracy (SVR-VLSM clusterwise *p* = 0.02; cluster size: 7.281 cm^3^; center of mass MNI coordinates: −56, −42.6, 6.5; Figure 4). No significant disconnections were identified related to the relative reduction of oral real word reading accuracy.

#### Pseudoword contrasts by OP mapping

**Figure 5.**
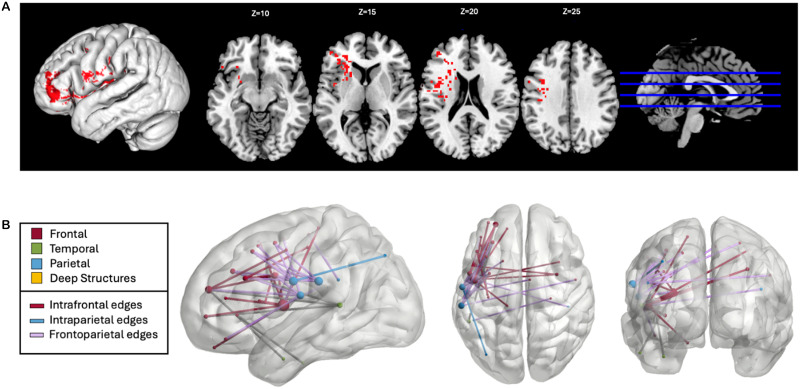
Lesion-symptom mapping for 1M + MM pseudowords controlling for 0M pseudowords. (A) Voxelwise lesion-symptom mapping results at *p* < 0.005 and clusterwise familywise error *p* < 0.05. (B) Connectome lesion-symptom mapping results show all significant edges color-coded by lobe, regardless of hemisphere. Edges that are not intrafrontal, intraparietal, or frontoparietal are shown in gray. Node size relates to the number of significant edges at that node.

The first contrast of pseudoword types aimed to identify lesions associated with loss of the ability to use learned OP mappings for sublexical reading. Lesions to a cluster extending from the anterior IFG, through the external capsule, and into the ventral precentral gyrus resulted in relative reduction of accuracy on pseudowords with body-level OP mappings (MM + 1M), controlling for those without learned mappings in English (0M) (SVR-VLSM clusterwise *p* = 0.01; cluster size: 8.109 cm^3^; center-of mass MNI coordinates: −37.7, 9.6, 12.4; Figure 5A). CLSM revealed 36 significant disconnections associated with reduced MM and 1M pseudoword reading (*p* < 0.05; see Figure 5B and Supplemental Table S1). Of these 36 disconnections, 14 connected LH frontal and parietal regions, 9 were LH intrafrontal connections, 5 were LH frontotemporal connections, and 7 were interhemispheric connections. The regions associated with the highest number of significant disconnections included the MFG (19 connections), the precentral gyrus (15), the postcentral gyrus (11), and the SMG (7).

**Figure 6.**
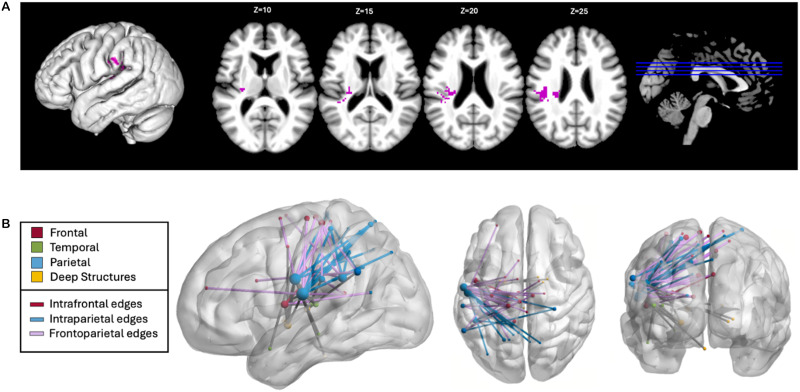
Lesion symptom mapping for MM Pseudowords controlling for 1M pseudowords. (A) Trending voxelwise lesion-symptom mapping results at *p* = 0.058 and clusterwise familywise error *p* < 0.05. (B) Connectome lesion-symptom mapping results show all significant edges color-coded by lobe, regardless of hemisphere. Edges that are not intrafrontal, intraparietal, or frontoparietal are shown in gray. Node size relates to the number of significant edges at that node.

Next, we aimed to identify lesions that result in impaired reading of pseudowords with multiple plausible OP mappings compared to those with single consistent mappings in English. Lesions to the anterior regions of the SMG were related to a relative reduction of MM pseudowords accuracy controlling for 1M accuracy but this result did not reach statistical significance (SVR-VLSM clusterwise *p* = 0.058, cluster size: 3.625 cm^3^; center-of mass MNI coordinates: −35.6, −26.7, 20.2; Figure 6A). CLSM revealed 55 significant disconnections associated with deficits in MM pseudowords (*p* < 0.05; see Figure 6B and Supplemental Table 1). These disconnections were primarily intraparietal and posterior frontoparietal in nature, including 14 intrafrontal, 13 frontoparietal, 8 parietal-subcortical, 3 frontosubcortical, 2 temporoparietal, 1 frontotemporal, and 14 interhemispheric connections. Regions most frequently implicated included the postcentral gyrus (36 disconnections), precentral gyrus (9 disconnections), and SMG (13 disconnections).

Finally, we aimed to identify lesions associated with specific deficits in sublexical reading without learned OP mappings of orthographic bodies. No significant lesions or disconnections were identified for the third sublexical contrast, the relative reduction of reading pseudowords without learned mappings (0M), controlling for pseudowords with learned mappings (MM, 1M).

## DISCUSSION

The primary aim of this study was to dissect the role of learned OP mappings in the sublexical route for reading. Our results clarify the distinction between the neuroanatomical bases of lexical and sublexical reading and affirms the role of multigraphemic learned OP mappings at the level of the orthographic body in sublexical reading. Depending on the nature of the orthographic body, reading an unfamiliar word relies on different subnetworks of processors within the broader frontoparietal sublexical reading network. These findings motivate further research into the mechanisms that underlie these subprocesses and the clinical relevance of deficits in applying learned OP mappings in alexia.

### Lesion Evidence for Sublexical and Lexical Routes for Reading in the Brain

Cognitive models of reading posit distinct processing routes for lexical and sublexical reading, particularly emphasizing that pseudowords, due to their lack of lexical content, are preferentially processed through the sublexical pathway (Coltheart et al., 2001; Perry et al., 2010; Plaut et al., 1996). Building upon prior activation and lesion studies suggesting that these reading pathways map onto distinct, distributed neural networks (Dickens et al., 2019; Martin et al., 2015; Price, 2012; Turkeltaub et al., 2002), our application of SVR-LSM to a large cohort of LHS survivors with variable reading impairments provides additional evidence that sublexical decoding of OP relies predominantly on dorsal perisylvian regions, while lexical processing is more dependent on ventral areas surrounding the superior temporal gyrus.

The syndrome of phonological alexia, which is defined based on the presence of pseudoword reading deficits, has been associated with large and variable lesions throughout perisylvian regions (Rapcsak et al., 2009; Ripamonti et al., 2014). In two prior LSM studies, our group examined phonological reading deficits based not on syndromic classification but rather on the lexicality effect as a continuous measure, as we do here (Dickens et al., 2019, 2021). These studies found that lesions of ventral precentral gyrus and SMG resulted in phonological reading deficits, albeit with different characteristics. Ventral precentral gyrus lesions were associated with isolated difficulties with pseudoword reading, while SMG lesions were associated with preferential deficits on pseudowords along with milder deficits on matched real words. The participants for one of these studies partly overlapped with the participants examined here (Dickens et al., 2021), but both of the previous studies used a different stimulus set: 20 short pseudowords (3–5 letters), primarily with 1M bodies, and a set of real words differing by one letter. The current findings using a more complex set of pseudoword stimuli provide a replication of the main findings from these prior studies implicating the ventral precentral gyrus and SMG in sublexical reading. The convergence of these findings with the 22 SMG-related disconnections identified in our CLSM analyses underscores the role of the SMG as both a key region and integrative node within the reading network.

The frontoparietal and intraparietal disconnections observed in our CLSM analysis implicate the wider dorsal processing network highlighted in prior research on sublexical reading (Aboud et al., 2016; Jobard et al., 2003; Taylor et al., 2013). Given that this analysis controls for accuracy in real word reading, the involvement of these regions is not solely related to processes such as letter recognition or speech production that are essential for all reading irrespective of lexical content. Consistent with the primary systems hypothesis (Woollams et al., 2018), our results identify disconnections involving regions subserving speech processing, including articulatory (left precentral gyrus), auditory (planum temporale), and somatosensory (ventral postcentral and supramarginal gyri) processing structures. Significant connections between the left postcentral gyrus, left SMG, left precentral gyrus, and left STS further align with the anatomy described in speech processing models (Bohland et al., 2010; Tourville & Guenther, 2011). The CLSM results implicated several nodes along the left MFG and their connections to parietal regions, largely the SMG. These connections with the MFG may be responsible for aspects of executive function such as working memory and attentional control which contribute to performance on complex phonological tasks like oral pseudoword reading (Deschamps et al., 2014; Kim, 2020).

Regarding the lexicosemantic route, we found that lesions to the lateral temporal regions, centered on the superior temporal sulcus, cause a diminished lexicality advantage during oral reading (i.e., a reduction in the typical advantage for reading words compared to pseudowords). This finding corroborates previous functional imaging research localizing lexicosemantic processing to a ventral word reading pathway including the MTG (Moore-Parks et al., 2010; Whitney et al., 2011). It also extends and aligns with prior lesion evidence identifying lesions to the posterior half of the MTG, extending into adjacent middle occipital gyrus resulting in regularization errors (Binder et al., 2016).

The lexicality advantage for reading observed in both accuracy and response duration measures, can relate either to the use of whole word orthographic and phonological representations or to the use of semantic content to support word reading (Patterson & Lambon Ralph, 1999). To clarify the mechanisms underlying lexicosemantic reading, and perhaps to isolate subregions involved in application of different types of lexical knowledge for reading, future research should employ contrasts that assess factors such as imageability, frequency, part of speech, or morphosyntactic features. A relative sparing of real word reading compared to pseudoword reading after lesions to the SMG and frontoparietal disconnections as found in our initial results suggests that the lexical pathway for reading does not critically pass through parietal regions. Instead, as suggested in some models of speech processing, the lexical pathway for reading may proceed anteriorly through the temporal lobe into inferior frontal cortex (Hickok, 2012). In this case, oral word reading may rely on direct connections between lexical representations in temporal cortex and articulatory plans for whole words in inferior frontal cortex.

### Role of OP Mappings in Sublexical Reading

Our findings provide key insights into the mechanisms underlying sublexical reading by affirming the crucial role of learned OP mappings at the level of the orthographic body. Behaviorally, pseudowords containing orthographic bodies that are present in English words were read more accurately than those without analogous orthographic bodies in both LHS and control participants. This pattern demonstrates that readers can effectively apply familiar multigraphemic OP mappings to novel stimuli.

Among control participants, this effect was also evident in response duration such that pseudowords with familiar orthographic bodies and a single plausible OP mapping were read more quickly than other pseudoword types, reflecting facilitation from learned OP patterns. In contrast, response duration increased when multiple mappings introduced ambiguity, or when no familiar mappings were available. This finding aligns with prior evidence that competition among possible pronunciations slows reading, even when accuracy is unaffected (Glushko, 1979; Taraban & McClelland, 1987). Unlike traditional reaction time measures, which index the latency to initiate a response, response duration reflects the total time spent on an item before advancing to the next trial, encompassing processing, articulation, and any delays related to doubt about the accuracy of the response. As such, it may offer a more comprehensive and clinically accessible metric of reading performance since spoken response times are difficult to measure in clinical practice. Given the relatively small number of items per condition and the low accuracy of many stroke participants, it was not feasible to use the response duration measure to assess the performance of the current stroke cohort. Future studies using a larger number of trials per condition in milder populations may help to detect additional lesion effects that may have been missed here because of this limitation.

Our LSM findings revealed involvement of the ventral precentral and post central gyri, SMG, anterior left IFG, and a network of frontoparietal regions and connections in reading pseudowords with learned body-level OP mappings as compared to pseudowords without such mappings. The findings of all the analyses collectively implicate the postcentral and precentral gyri as highly interconnected regions supporting pseudoword reading relative to real word reading, particularly for pseudowords with learned body-level OP mappings. While these regions are well-known for their involvement in motor articulatory and somatosensory representations from models of speech production (Bohland et al., 2010; Hickok, 2012; Tourville & Guenther, 2011; Walker & Hickok, 2016), our results suggest a more nuanced role in reading. In a prior LSM study, we found that the pseudoword reading deficit caused by lesions to these structures was specifically associated with motor phonological impairments, which caused an isolated pseudoword reading deficit with no effect on matched real words (Dickens et al., 2021). Here, we found that ventral sensorimotor regions were particularly important for reading pseudowords with learned body-level OP mappings. The contribution of these structures to pseudoword reading cannot be explained purely by speech production deficits, since oral word reading performance was covaried in both the prior and the current study. Instead, the involvement of the precentral and postcentral gyri suggests that learning to read may co-opt speech motor and somatosensory units for functions beyond simple articulation (Dickens et al., 2021). Specifically, since the reading of pseudowords with learned OP mappings was disrupted after lesions to, and disconnections from, these regions, the precentral and postcentral gyri are likely involved in storing, accessing, or assembling learned OP mappings. This corresponds with the primary roles of these regions in storing and assembling motor plans for speech production. Like OP mappings, speech motor plans must link to sensory representations and are stored at various grain sizes depending on the frequency of use (Tourville & Guenther, 2011). Similar computations may underlie selection and assembly of speech motor plans and OP mappings at various grain sizes. As such, learning to read may coopt sensorimotor regions evolved for speech production to store and assemble learned OP mappings for reading unfamiliar words.

Our main lexicality results implicated the SMG in pseudoword reading overall, suggesting that the SMG plays a central role in sublexical reading with and without learned body-level OP mappings. This aligns with prior fMRI and transcranial magnetic stimulation studies that have identified the SMG as a critical region involved with several types of phonological processing (Jobard et al., 2003; Oberhuber et al., 2016; Vigneau et al., 2006), including phonological working memory (Deschamps et al., 2014) and phonological decoding during reading (Jobard et al., 2003). A prior LSM study on lexicality effects in a partly overlapping cohort using different reading items implicated the SMG in sensorimotor translation processes important for both speech and reading (Dickens et al., 2021). In this prior study, sensorimotor translation deficits, as reflected by poor pseudoword repetition, related to alexia that preferentially affected pseudowords, but also real words with regular spellings, albeit to a lesser degree. Notably, in the current study, the MM controlling for 1M SVR-VLSM analysis also identified the SMG, suggesting it may be especially important for sublexical reading when multiple body-level OP mappings are available. The corresponding CLSM analysis implicated the left postcentral gyrus, left precentral gyrus, left SMG, and a broader network of intraparietal connections. This finding may indicate an increased reliance on learned OP mappings in the precentral and postcentral gyri, and phonological processing and working memory in the SMG and other posterior parietal regions when deciding between multiple plausible pronunciations for an unfamiliar word. Overall, it is clear that the SMG plays a central role in sublexical reading, but its exact role remains enigmatic. It is possible that subregions of the SMG perform different specific processes important to different aspects of phonology and sublexical reading, or that the SMG performs a computation that underlies various specific phonological operations and renders it important for sublexical reading in multiple ways. Disentangling the specific contributions of the SMG to sublexical reading and phonology should be a focus of future research.

The anterior IFG, which was implicated in reading pseudowords with learned OP mappings here (1M + MM pseudowords), has been previously implicated in selection and control processes, often linked to semantic retrieval (Binder et al., 2005; Mechelli et al., 2007; Poldrack et al., 1999; Taylor et al., 2013). The left IFG has been directly related to phonological processing, though typically in more dorsal and posterior regions such as the pars opercularis (Vigneau et al., 2006). In this context, one possible interpretation of the anterior IFG result here is that the IFG is engaged in suppressing potential lexicalizations or in drawing analogies between pseudowords and known real words or mappings. Alternatively, the IFG could provide a facilitatory control signal to boost OP mapping representations in sensorimotor regions or the SMG. This suggests that the anterior IFG may contribute to selecting appropriate phonological representations through either of these mechanisms. However, this interpretation is complicated by our second sublexical contrast, which did not implicate the anterior IFG in pseudowords with multiple OP mappings compared to those with a single learned OP mapping. If the IFG’s primary function were indeed in selecting relevant sublexical representations, we would expect greater reliance specifically in the sublexical contrast isolating pseudowords with multiple plausible pronunciations to select between (i.e., MM pseudowords). Therefore, our results suggest the IFG’s role is not in selecting representations during sublexical reading, but in either suppressing potential lexicalizations or accessing relevant lexical knowledge. These findings call for further investigation into the anterior IFG’s mechanistic role in sublexical processing.

The CLSM results also implicated connections to the left MFG in reading pseudowords with OP mappings versus those without. The MFG has been implicated in representations of both orthographic and phonological information (Graves et al., 2023; Zhao et al., 2017) as well as in attentional and working memory demands related to language and phonological processing (Barbeau et al., 2023; Curtis, 2006; Mottaghy et al., 2003). Alternatively, studies of the gray matter correlates of working memory have suggested that the working memory loop for phonological content is more reliant on the SMG and posterior IFG while the anterior IFG, MFG, and angular gyrus (AG) are more related to semantic working memory (Horne et al., 2023; Martin & He, 2004; Paulesu et al., 1993; Vigneau et al., 2006). This framework allows for the possibility that mechanisms usually employed for semantic working memory processes in frontal regions may be recruited specifically when reading pseudowords with learned OP mappings, perhaps due to their associations with lexical items. Alternatively, the MFG may have more multimodal, general processing capacities (Diachek et al., 2020). A number of interhemispheric connections were also implicated in reading using learned OP mappings. These connections involve subcortical and medial regions of the right hemisphere rather than regions homotopic to canonical language processing regions in the LH, again suggesting a general cognitive contribution to applying learned OP mappings while reading unfamiliar words.

This study employed both voxel-based and connectome-based lesion-symptom mapping to begin disentangling the relative contributions of focal cortical lesions and network disconnections to deficits in sublexical reading. While connectome-based approaches can extend inferences beyond the lesioned cortex, they also introduce challenges in attributing deficits to either direct regional damage or disrupted structural interactions. In this context, we observed disconnections involving regions not implicated by VLSM alone, suggesting that network-level disruption contributes to impaired sublexical processing. Moreover, the presence of distinct disconnection patterns—such as intraparietal versus frontoparietal pathways converging on the SMG—underscores that these effects cannot be fully explained by cortical damage alone. To enhance interpretability, we used a conservative binary disconnection approach, defining edges as lesioned only when absent in stroke participants and reliably present in all controls. This method anchors network disruption to lesion-induced tract absence, while minimizing sensitivity to interindividual variability in connection strength. Nonetheless, fully dissociating the effects of gray matter lesions and white matter disconnection remains complex. Future work may benefit from incorporating graded measures of connectivity, such as fiber density or composite indices, to capture partial damage and examine dose-dependent effects of structural degradation.

Although our contrasts identified behavioral differences between pseudoword types and lesion correlates for reading pseudowords with learned body-level OP mappings (1M and MM), we did not observe significant lesion correlates for 0M pseudowords when controlling for the other pseudoword types. Processes supporting 0M reading—such as smaller grain mapping and unit assembly—may also underlie performance across pseudoword types, limiting detectable dissociations in neural data. Future work may benefit from exploring 0M reading with larger stimulus sets or alternative methods that can better capture shared processing.

Additionally, while most participants in our sample were in the chronic stage of recovery (i.e., ≥6 mo post-stroke), a small number were still in the subacute phase. While stroke chronicity was not significantly associated with any of the primary behavioral measures examined here, and thus was not included as a covariate in the present analyses, we acknowledge this as a limitation. Future research with larger samples could more directly examine whether stage of recovery modulates patterns of reading performance or lesion-deficit associations.

Clinically, these results highlight the importance of developing a more nuanced diagnostic scheme for alexia. While a specific deficit of pseudoword reading relative to real word reading is the hallmark symptom of phonological alexia, this diagnosis encompasses a variety of specific processing deficits resulting in the common phenotype of impaired pseudoword reading. Like our prior study describing two types of phonological reading deficits related to different phonological subprocesses (Dickens et al., 2021), this study provides additional evidence that alexia may be better described based on specific process-level deficits. Further, these results highlight the potential of targeted rehabilitation approaches that leverage preserved neural circuits for phonological and lexical processing. For example, interventions focusing on retraining learned body-level OP mappings or compensating for specific disconnections within the reading network could be tailored to individual lesion profiles. Future research is needed to explore how lesion-induced network disruptions influence dynamic recovery processes to further refine neurorehabilitation strategies.

## ACKNOWLEDGMENTS

For their valuable contributions we thank our participants and, in alphabetical order, our data collectors: Elizabeth Dvorak, Trini Kelly, Elizabeth Lacey, Alycia Laks, Sachi Paul, and Candace van der Stelt.

## FUNDING INFORMATION

Sara M. Dyslin, National Institute on Deafness and Other Communication Disorders (https://dx.doi.org/10.13039/100000055), Award ID: T32DC019481. Sara M. Dyslin, National Institute on Deafness and Other Communication Disorders (https://dx.doi.org/10.13039/100000055), Award ID: F31DC022513. Andrew T. DeMarco, National Institute on Deafness and Other Communication Disorders (https://dx.doi.org/10.13039/100000055), Award ID: K99DC018828. Ryan Staples, National Center for Advancing Translational Sciences (https://dx.doi.org/10.13039/100006108), Award ID: TL1TR001431. J. Vivian Dickens, National Institute on Deafness and Other Communication Disorders (https://dx.doi.org/10.13039/100000055), Award ID: F30DC018215. Peter E. Turkeltaub, National Institute on Deafness and Other Communication Disorders (https://dx.doi.org/10.13039/100000055), Award ID: R01DC014960. Peter E. Turkeltaub, National Institute on Deafness and Other Communication Disorders (https://dx.doi.org/10.13039/100000055), Award ID: R01DC020446.

## AUTHOR CONTRIBUTIONS

**Sara M. Dyslin**: Conceptualization; Formal analysis; Investigation; Methodology; Visualization; Writing – original draft; Writing – review & editing. **Andrew T. DeMarco**: Conceptualization; Data curation; Formal analysis; Methodology; Software; Supervision; Writing – review & editing. **Ryan Staples**: Formal analysis; Writing – review & editing. **J. Vivian Dickens**: Conceptualization; Data curation; Investigation; Methodology; Writing – review & editing. **Sarah F. Snider**: Data curation; Investigation; Writing – review & editing. **Rhonda Friedman**: Conceptualization; Writing – review & editing. **Peter E. Turkeltaub**: Conceptualization; Formal analysis; Funding acquisition; Methodology; Project administration; Supervision; Writing – review & editing.

## DATA AND CODE AVAILABILITY STATEMENT

The pipeline used for warping lesion masks to the Clinical Toolbox Older Adult Template using Advanced Normalization Tools (ANTs) is available at https://stnava.github.io/ANTs/. The Lausanne atlas used to identify edges of the structural connectome is available at https://github.com/mattcieslak/easy_lausanne. The multivariate lesion-symptom mapping MATLAB toolbox is described in DeMarco and Turkeltaub (2018) and available at https://github.com/atdemarco/svrlsmgui. Pseudoword stimuli are available in Supplemental Materials, and real word stimuli are available in the supplemental materials of Staples et al. (2025). De-identified behavioral and imaging data are available at https://github.com/crlabgeorgetown/DyslinNOL2025. Further inquiries about data or analysis materials can be directed to the corresponding author.

## Supplementary Material



## TECHNICAL TERMS

Orthography-to-phonology (OP) mapping: Learned correspondence between written orthographic units and their pronunciations.
Orthographic body: The portion of a one-syllable word consisting of the vowel and all following letters, used to determine spelling–sound patterns (e.g., -ead in “head” and “bead”).
Lesion-symptom mapping (LSM): A statistical technique that allows researchers to associate patterns of brain damage with behavioral performance.
