# Supplementary material for: Dissecting the Sublexical Route for Reading: Frontal and Parietal Networks Support Learned Orthography-to-Phonology Mappings
Source: Neurobiol Lang (Camb). 2026 Jan 28;7:NOL.a.216. doi: 10.1162/NOL.a.216 (PMC12978678; doi:10.1162/NOL.a.216)
Supplement: Supplementary file 1 [file nol-07-216-s001.pdf]

Supplemental Materials

Supplemental Table 1: SVR-CLSM results for oral pseudoword reading accuracy in contrasts 1a, 2a, and 2b.

Contrast 1a: Pseudoword vs real word reading

	Edge	SVR-β	MNI 1	MNI 2
Intra-Frontal	Left pars opercularis <> Left superior frontal gyrus	7.6236	-38, 15, 12	-6, -1, 60
	Left rostral middle frontal gyrus <> Left precentral gyrus	8.4137	-40, 39, 14	-51, 4, 30
Fronto-Parietal	Left rostral middle frontal gyrus <> Left postcentral gyrus	8.6913	-44, 25, 28	-58, -9, 26
	Left superior frontal gyrus <> Left postcentral gyrus	7.6083	-18, 1, 67	-58, -9, 26
	Left caudal middle frontal gyrus <> Left postcentral gyrus	9.1479	-32, 4, 53	-58, -9, 26
	Left precentral gyrus <> Left postcentral gyrus	9.2243	-47, -4, 48	-54, -14, 16
	Left posterior cingulate gyrus <> Left postcentral gyrus	8.289	-5, -15, 33	-54, -14, 16
	Left superior frontal gyrus <> Left SMG	7.6036	-18, 1, 67	-59, -27, 26
	Left caudal middle frontal gyrus <> Left SMG	10	-32, 4, 53	-59, -27, 26
	Left rostral middle frontal gyrus <> Left SMG	9.5239	-44, 25, 28	-47, -41, 27
	Left precentral gyrus <> Left SMG	8.6792	-51, 4, 30	-47, -41, 27
	Left rostral middle frontal gyrus <> Left SMG	7.6578	-44, 25, 28	-58, -51, 31
	Left rostral middle frontal gyrus <> Left SMG	8.0818	-40, 39, 14	-58, -51, 31
	Left rostral middle frontal gyrus <> Left SMG	7.8649	-26, 51, 20	-58, -51, 31
	Left superior frontal gyrus <> Left SMG	8.0763	-19, 16, 51	-58, -51, 31
	Left rostral middle frontal gyrus <> Left SMG	8.3467	-40, 39, 14	-40, -38, 38
	Left rostral middle frontal gyrus <> Left inferior parietal lobule	8.9196	-44, 25, 28	-39, -56, 23
	Left precentral gyrus <> Left inferior parietal lobule	9.0923	-47, -2, 9	-35, -74, 44
Fronto-Temporal	Left superior frontal gyrus <> Left STS	8.8404	-6, -1, 60	-52, -42, 9
	Left superior frontal gyrus <> Left STS	8.5476	-18, 1, 67	-52, -42, 9
	Left caudal middle frontal gyrus <> Left STS	9.204	-32, 4, 53	-52, -42, 9
	Left precentral gyrus <> Left STS	8.8588	-40, -13, 33	-52, -42, 9
	Left precentral gyrus <> Left superior temporal gyrus	8.598	-40, -13, 33	-57, -20, -1
Intra-Parietal	Left paracentral gyrus <> Left postcentral gyrus	8.02	-10, -28, 49	-54, -14, 16
	Left postcentral gyrus <> Left postcentral gyrus	8.8231	-16, -36, 72	-54, -14, 16
	Left postcentral gyrus <> Left postcentral gyrus	8.555	-27, -33, 60	-54, -14, 16
	Left postcentral gyrus <> Left SMG	8.3621	-41, -31, 55	-59, -27, 26
	Left postcentral gyrus <> Left SMG	8.1725	-44, -20, 48	-59, -27, 26
	Left postcentral gyrus <> Left SMG	7.9903	-16, -36, 72	-47, -41, 27
	Left postcentral gyrus <> Left SMG	8.0567	-27, -33, 60	-47, -41, 27
	Left postcentral gyrus <> Left SMG	9.2603	-58, -9, 26	-47, -41, 27
	Left SMG <> Left SMG	9.0062	-59, -27, 26	-47, -41, 27
	Left postcentral gyrus <> Left SMG	8.5844	-58, -9, 26	-40, -38, 38
	Left postcentral gyrus <> Left superior parietal gyrus	7.5703	-58, -9, 26	-21, -45, 60
	Left postcentral gyrus <> Left superior parietal gyrus	8.5623	-58, -9, 26	-35, -45, 56
	Left postcentral gyrus <> Left superior parietal gyrus	7.7011	-54, -14, 16	-23, -55, 41
	Left postcentral gyrus <> Left inferior parietal lobule	8.457	-58, -9, 26	-39, -56, 23
	Left postcentral gyrus <> Left inferior parietal lobule	8.6793	-58, -9, 26	-35, -74, 44
	Left postcentral gyrus <> Left inferior parietal lobule	8.7004	-54, -14, 16	-35, -74, 44
	Left SMG <> Left inferior parietal lobule	8.6925	-59, -27, 26	-35, -74, 44
	Left SMG <> Left precuneus gyrus	8.5728	-47, -41, 27	-5, -53, 58
Parietal-Subcortical	Left postcentral gyrus <> Left thalamus proper	8.9873	-58, -9, 26	-14, -17, 6
	Left postcentral gyrus <> Left thalamus proper	8.5634	-54, -14, 16	-14, -17, 6
	Left SMG <> Left thalamus proper	8.3573	-59, -27, 26	-14, -17, 6
	Left postcentral gyrus <> Left hippocampus	9.087	-54, -14, 16	-24, -25, -11
	Left postcentral gyrus <> Left brainstem	8.3836	-54, -14, 16	0, -29, -26
	Left isthmus cingulate gyrus <> Left SMG	8.2819	-9, -42, 16	-47, -41, 27
Temporo-Parietal	Left postcentral gyrus <> Left STS	8.1939	-16, -36, 72	-52, -42, 9
	Left postcentral gyrus <> Left STS	9.0144	-58, -9, 26	-52, -42, 9
Inter-hemispheric	Right posterior cingulate <> Left postcentral gyrus	8.4574	7, -25, 34	-54, -14, 16
	Right caudate <> Left postcentral gyrus	8.0688	14, 1, 9	-54, -14, 16
	Right putamen <> Left postcentral gyrus	8.4343	23, -1, -2	-54, -14, 16
	Right pallidum <> Left SMG	8.682	19, -4, -4	-58, -51, 31

Contrast 2a: Pseudoword reading: OP mappings vs no mappings (1M+MM>0M)

	Edge	SVR-β	MNI 1	MNI 2

Intra-Frontal	Left rostral middle frontal gyrus <> Left precentral gyrus	7.2845	-44, 25, 28	-33, -19, 50
	Left rostral middle frontal gyrus <> Left precentral gyrus	7.6014	-26, 51, 20	-47, -4, 48
	Left rostral middle frontal gyrus <> Left precentral gyrus	8.1457	-44, 25, 28	-51, 4, 30
	Left rostral middle frontal gyrus <> Left precentral gyrus	7.6202	-26, 51, 20	-51, 4, 30
	Left lateral orbitofrontal gyrus <> Left precentral gyrus	7.7413	-15, 47, -21	-47, -2, 9
	Left pars opercularis gyrus <> Left precentral gyrus	7.6268	-38, 15, 12	-47, -2, 9
	Left rostral middle frontal gyrus <> Left precentral gyrus	8.103	-40, 39, 14	-47, -2, 9
	Left rostral middle frontal gyrus <> Left precentral gyrus	8.1356	-33, 53, 1	-47, -2, 9
	Left precentral gyrus <> Left precentral gyrus	7.341	-15, -27, 64	-47, -2, 9
Fronto-Parietal	Left superior frontal gyrus <> Left postcentral gyrus	7.227	-19, 16, 51	-58, -9, 26
	Left caudal middle frontal gyrus <> Left postcentral gyrus	10	-35, 20, 47	-58, -9, 26
	Left caudal middle frontal gyrus <> Left postcentral gyrus	9.1106	-32, 4, 53	-58, -9, 26
	Left precentral gyrus <> Left postcentral gyrus	8.317	-51, 4, 30	-58, -9, 26
	Left precentral gyrus <> Left postcentral gyrus	7.23	-47, -2, 9	-58, -9, 26
	Left pars orbitalis gyrus <> Left postcentral gyrus	8.4273	-41, 39, -15	-54, -14, 16
	Left rostral middle frontal gyrus <> Left postcentral gyrus	7.4251	-29, 34, 33	-54, -14, 16
	Left rostral middle frontal gyrus <> Left postcentral gyrus	7.2846	-26, 51, 20	-54, -14, 16
	Left precentral gyrus <> Left postcentral gyrus	7.4471	-51, 4, 30	-54, -14, 16
	Left rostral middle frontal gyrus <> Left SMG	8.0798	-44, 25, 28	-59, -27, 26
	Left rostral middle frontal gyrus <> Left SMG	7.1838	-29, 34, 33	-59, -27, 26
	Left caudal middle frontal gyrus <> Left SMG	7.6613	-35, 20, 47	-59, -27, 26
	Left caudal middle frontal gyrus <> Left SMG	7.7638	-34, 7, 36	-59, -27, 26
	Left caudal middle frontal gyrus <> Left SMG	9.3195	-32, 4, 53	-59, -27, 26
Fronto-Temporal	Left pars triangularis gyrus <> Left fusiform gyrus	7.5713	-40, 31, 2	-34, -16, -32
	Left rostral middle frontal gyrus <> Left middle temporal gyrus	7.0966	-26, 51, 20	-53, -2, -29
	Left rostral middle frontal gyrus <> Left STS	8.5535	-29, 34, 33	-52, -42, 9
	Left rostral middle frontal gyrus <> Left STS	8.3632	-26, 51, 20	-52, -42, 9
	Left rostral middle frontal gyrus <> Left STS 1	7.2402	-33, 53, 1	-52, -42, 9
Intra-Parietal	Left postcentral gyrus <> Left inferior parietal lobule	6.9499	-58, -9, 26	-35, -74, 44
Inter-hemispheric	Right caudal middle frontal gyrus <> Left pars opercularis	7.2361	33, 3, 38	-47, 11, 11
	Right caudal middle frontal gyrus <> Left precentral gyrus	7.7506	33, 3, 38	-47, -2, 9
	Right caudal middle frontal gyrus <> Left precentral gyrus	7.7929	35, 15, 54	-47, -2, 9
	Right superior frontal gyrus <> Left postcentral gyrus	6.8891	7, -2, 56	-54, -14, 16
	Right precentral gyrus <> Left SMG	7.4528	51, -2, 33	-59, -27, 26
	Right precentral gyrus <> Left SMG	7.6892	27, -20, 59	-47, -41, 27
	Right SMG <> Left precentral gyrus	7.2248	44, -28, 22	-47, -2, 9

Contrast 2b: Pseudoword reading: Multiple OP mappings vs 1 OP mapping (MM>1M)

	**Edge**	**SVR-β**	**MNI1**	**MNI2**
Fronto-Parietal	Left superior frontal gyrus <> Left postcentral gyrus	7.8377	-6, -1, 60	-58, -9, 26
	Left superior frontal gyrus <> Left postcentral gyrus	7.2814	-18, 1, 67	-58, -9, 26
	Left posterior cingulate gyrus <> Left postcentral gyrus	8.544	-5, -15, 33	-58, -9, 26
	Left rostral middle frontal gyrus <> Left postcentral gyrus	6.9117	-26, 51, 20	-54, -14, 16
	Left precentral gyrus <> Left postcentral gyrus	7.3714	-15, -27, 64	-54, -14, 16
	Left precentral gyrus <> Left postcentral gyrus	7.9729	-31, -17, 67	-54, -14, 16
	Left posterior cingulate gyrus <> Left postcentral gyrus	8.9768	-5, -15, 33	-54, -14, 16
	Left isthmus cingulate gyrus <> Left postcentral gyrus	7.8818	-9, -42, 16	-54, -14, 16
	Left precentral gyrus <> Left SMG	7.3852	-15, -27, 64	-59, -27, 26
	Left isthmus cingulate gyrus <> Left SMG	7.9795	-9, -42, 16	-59, -27, 26
	Left precentral gyrus <> Left SMG	7.5955	-15, -27, 64	-47, -41, 27
	Left posterior cingulate gyrus <> Left SMG	8.1485	-5, -15, 33	-47, -41, 27
	Left caudal anterior cingulate gyrus <> Left SMG	8.2253	-5, 17, 25	-58, -51, 31
	Left precentral gyrus <> Left precuneus	7.1546	-47, -2, 9	-14, -60, 17
Fronto-Subcortical	Left precentral gyrus <> Left insula	7.8832	-47, -2, 9	-34, -20, 9
	Left precentral gyrus <> Left pallidum	7.7726	-47, -4, 48	-19, -4, -5
	Left precentral gyrus <> Left pallidum	7.1736	-47, -2, 9	-19, -4, -5
Fronto-temporal	Left precentral gyrus <> Left superior temporal gyrus	8.1252	-31, -17, 67	-46, 8, -21
Intra-parietal	Left paracentral gyrus <> Left postcentral gyrus	8.0689	-4, -23, 68	-58, -9, 26
	Left paracentral gyrus <> Left postcentral gyrus	7.5615	-10, -28, 49	-54, -14, 16
	Left paracentral gyrus <> Left SMG	7.8961	-4, -23, 68	-58, -51, 31
	Left postcentral gyrus <> Left postcentral gyrus	8.4215	-27, -33, 60	-54, -14, 16
	Left postcentral gyrus <> Left SMG	7.5563	-27, -33, 60	-47, -41, 27
	Left postcentral gyrus <> Left superior parietal gyrus	8.4515	-54, -14, 16	-21, -45, 60
	Left postcentral gyrus <> Left superior parietal gyrus	7.6421	-58, -9, 26	-19, -60, 63
	Left postcentral gyrus <> Left superior parietal gyrus	7.9448	-54, -14, 16	-19, -60, 63

	Left postcentral gyrus <> Left inferior parietal lobule	7.2972	-54, -14, 16	-35, -74, 44
	Left postcentral gyrus <> Left precuneus	8.3537	-54, -14, 16	-5, -53, 58
	Left postcentral gyrus <> Left precuneus	8.8436	-54, -14, 16	-11, -45, 44
	Left SMG <> Left precuneus	8.0909	-47, -41, 27	-11, -45, 44
	Left postcentral gyrus <> Left precuneus	7.6578	-54, -14, 16	-10, -55, 34
Parietal-Subcortical	Left postcentral gyrus <> Left thalamus proper	7.8301	-58, -9, 26	-14, -17, 6
	Left postcentral gyrus <> Left thalamus proper	7.4219	-54, -14, 16	-14, -17, 6
	Left SMG <> Left thalamus proper	6.973	-59, -27, 26	-14, -17, 6
	Left postcentral gyrus <> Left putamen	7.3957	-58, -9, 26	-23, -3, -2
	Left postcentral gyrus <> Left pallidum	8.6876	-58, -9, 26	-19, -4, -5
	Left postcentral gyrus <> Left pallidum	8.3363	-54, -14, 16	-19, -4, -5
	Left postcentral gyrus <> Left hippocampus	7.996	-58, -9, 26	-24, -25, -11
	Left postcentral gyrus <> Left brainstem	8.9339	-58, -9, 26	0, -29, -26
Temporo-parietal	Left postcentral gyrus <> Left transverse temporal gyrus	10	-58, -9, 26	-41, -23, 9
	Left postcentral gyrus <> Left transverse temporal gyrus	7.1277	-54, -14, 16	-41, -23, 9
Inter-hemispheric	Right posterior cingulate gyrus <> Left precentral gyrus	7.3793	7, -25, 34	-47, -2, 9
	Right precentral gyrus <> Left postcentral gyrus	8.1286	20, -15, 61	-54, -14, 16
	Right posterior cingulate gyrus <> Left postcentral gyrus	8.1897	7, -25, 34	-54, -14, 16
	Right superior frontal gyrus <> Left SMG	7.5391	15, -3, 69	-47, -41, 27
	Right paracentral gyrus <> Left postcentral gyrus	7.47	3, -33, 65	-58, -9, 26
	Right paracentral gyrus <> Left postcentral gyrus	8.4786	9, -17, 53	-54, -14, 16
	Right paracentral gyrus <> Left SMG	7.6716	9, -17, 53	-47, -41, 27
	Right postcentral gyrus <> Left postcentral gyrus	7.452	30, -30, 70	-58, -9, 26
	Right precuneus gyrus <> Left postcentral gyrus	7.8549	6, -62, 46	-58, -9, 26
	Right superior parietal gyrus <> Left postcentral gyrus	8.3933	16, -63, 65	-54, -14, 16
	Right postcentral gyrus <> Left SMG	6.7992	30, -30, 70	-58, -51, 31
	Right putamen <> Left postcentral gyrus	7.4415	23, -1, -2	-58, -9, 26
	Right caudate <> Left postcentral gyrus	8.4326	14, 1, 9	-54, -14, 16
	Right pallidum <> Left SMG	7.9951	19, -4, -4	-58, -51, 31

*SVR-β support vector regression beta coefficient. MNI 1 and 2 represent the Montreal Neurologic Institute coordinates for parcel centroids to the left and right of the ‘<>’. $P<0.0001$ for all edges. An edge consists of the connection between two Lausanne Atlas Scale 125 parcels.

Supplemental Table 2: Oral Pseudoword (PW) Reading Stimuli by Type

1M PW	MM PW	0M PW
bink	fost	bolg
broal	drost	broub
cout	clow	caup
cland	trow	clais
crast	chead	crilp
dife	gead	dofe
drate	grour	dronf
frout	vour	froum
hish	pown	hirn
meech	glown	maich
nart	slind	noug
pilt	glind	polb
prike	slear	preln
stend	plear	stilp
tace	gron	tuve
tarch	fron	talch
vage	stoth	veif
wribe	hoth	wrofe
yoon	spo	yoog
zask	vo	zomk
